# *Trichophyton rubrum* Phenotypic Virulence Factors in Mexican Strains

**DOI:** 10.3390/biology14060661

**Published:** 2025-06-07

**Authors:** Esther Conde-Cuevas, Rigoberto Hernández-Castro, Claudia Erika Fuentes-Venado, Roberto Arenas, María Guadalupe Frías-De-León, Gabriela Moreno-Coutiño, María Esther Ocharan-Hernández, Eunice D. Farfan-Garcia, Rodolfo Pinto-Almazán, Erick Martínez-Herrera

**Affiliations:** 1 Maestría en Ciencias de la Salud, Escuela Superior de Medicina, Instituto Politécnico Nacional, Plan de San Luis y Díaz Mirón, Ciudad de México 11340, Mexico; condeesther999@gmail.com; 2 Departamento de Ecología de Agentes Patógenos, Hospital General “Dr. Manuel Gea González”, Ciudad de México 14080, Mexico; rigo37@gmail.com; 3 Servicio de Medicina Física y Rehabilitación, Hospital General de Zona No 197, Texcoco 56108, Mexico; cefvenado@hotmail.com; 4 Sección de Estudios de Posgrado e Investigación, Escuela Superior de Medicina, Instituto Politécnico Nacional, Plan de San Luis y Díaz Mirón s/n, Col. Casco de Santo Tomas, Alcaldía Miguel Hidalgo, Ciudad de México 11340, Mexico; estherocharan@hotmail.com; 5 Sección de Micología, Hospital General “Dr. Manuel Gea González”, Tlalpan, Ciudad de México 14080, Mexico; rarenas98@hotmail.com (R.A.); gmorenocoutino@gmail.com (G.M.-C.); 6 Unidad de Investigación Biomédica, Hospital Regional de Alta Especialidad de Ixtapaluca, Servicios de Salud del Instituto Mexicano de Seguro Social para el Bienestar (IMSS-BIENESTAR), Carretera Federal México-Puebla Km 34.5, Ixtapaluca 56530, Mexico; magpefrias@gmail.com; 7 Bioquímica, Sección de Estudios de Posgrado e Investigación, Escuela Superior de Medicina, Instituto Politécnico Nacional, Plan de San Luis y Díaz Mirón, Ciudad de México 11340, Mexico; efarfang@ipn.mx; 8 Fundación Vithas, Grupo Hospitalario Vithas, 28043 Madrid, Spain; 9 Efficiency, Quality, and Costs in Health Services Research Group (EFISALUD), Galicia Sur Health Research Institute (IISGS), Servizo Galego de Saúde-Universidade de Vigo (UVIGO), 36310 Vigo, Spain

**Keywords:** *Trichophyton rubrum*, virulence factors, lipase, phospholipase, hemolysins, elastase

## Abstract

(1) Background: *T. rubrum* is the most important agent in *tinea pedis*, *tinea manuum*, *tinea cruris*, *tinea corporis*, and even in subcutaneous dermatophytosis. *T. rubrum* must overcome several obstacles to adhere, grow, and invade the host, for which their virulence factors are important. Previous studies have demonstrated the capability of *T. rubrum* strains to produce proteases, phospholipases, hemolysins, and elastases. The aim of this work was the genotypic identification of clinical isolates of *T. rubrum* to subsequently determine production of the main phenotypic virulence factors associated with this pathogen responsible for different types of dermatophytosis in Mexican patients. (2) Methods: Twenty samples of *T. rubrum* were obtained from different body parts of patients treated in the Mycology section. The colonies were transferred to specific agars to analyze the production of phenotypical virulence factors (lipase, phospholipase, hemolysin, and elastase). (3) Results: Almost all the strains of *T. rubrum* showed growth in the test culture medium. A significantly smaller size of the halo diameter of elastase (26.51 ± 11.95 mm) in comparison to lipase (59.51 ± 16.00 mm) and phospholipase (55.97 ± 19.60 mm) was measured. Additionally, a significantly reduced size of the halo diameter of hemolysin (42.01 ± 5.49 mm) was observed compared to lipase. When comparing the virulence factors, greater expression of lipase was observed, followed by phospholipase, hemolysins, and elastase. *T. rubrum* strains were classified as being between high and ultra-lipase producers; most of the strains were also considered low producers of phospholipase and hemolysins; and most of the strains (*n* = 13) were classified as non-producers of elastase. (4) Conclusions: Almost all the *T. rubrum* strains of the study were found to be ultra-producers of lipase, and low producers of hemolysins and phospholipases. Elastase was the least expressed virulence factor in these strains.

## Simple Summary

*Trichophyton rubrum* is a fungus that causes skin infections in humans. *T. rubrum* possesses several virulence factors, among them, lipase, phospholipase, hemolysin, and elastase, which allow it to bind to skin cells and alter their structure to penetrate, invade, and grow within them. In this study, we identified the virulence factors present in 20 *T. rubrum* strains obtained from patients in Mexico. We observed that most strains had highly active lipases, low-activity phospholipases and hemolysins, and almost inactive elastases. This means that in Mexico, lipase was the main virulence factor used by *T. rubrum* to infect patients, while elastase appeared to be the least.

## 1. Introduction

Dermatomycoses are common superficial fungal infections caused by keratin parasitic fungi of the skin, nails, and hair [1,2]. Formerly, dermatomycoses comprised three anamorph genera: *Trichophyton*, *Epidermophyton*, and *Microsporum* [3,4], although the most recent classification divides them into *Trichophyton*, *Epidermophyton*, *Nannizzia*, *Microsporum*, *Lophophyton*, *Arthroderma*, and *Ctenomyces* genera [5].

Dermatophytes are cosmopolite pathogens [6], responsible for 5% of the dermatologic consultations, and reports a worldwide prevalence of 20–25% [1,4,7]. In Mexico, dermatophytes are acknowledged for up to 80% of diagnosed fungal infections [8].

Depending on their host target, dermatophytes are classified as geophilic, zoophilic, and anthropophilic. In humans, the main geophilic agent is *Nannizzia gypsea* (*N. gypsea*); *Microsporum canis* (*M. canis*) leads to a zoophilic type, and *Trichophyton rubrum* (*T. rubrum*) causes anthropophilic [9,10] infection.

*T. rubrum* is the most prevalent isolated etiological agent in skin and nails. Also, *T. rubrum* has been recognized as the most important agent in *tinea pedis*, *tinea manuum*, *tinea cruris*, *tinea corporis*, and even subcutaneous dermatophytosis (Majochii granuloma) [4,11,12]. The most important method of transmission of *T. rubrum* is direct and indirect contact. Nonetheless, *T. rubrum* is not capable of invading some parts of the body such as hair and follicles. It has a global distribution, regardless of race, age, and gender, mainly because human migration and traveling have contributed to epidemiological changes in global dissemination [13,14]. In Mexico, the second-most frequent dermatophyte is *Trichophyton tonsurans* (*T. tonsurans*), followed by *Trichophyton mentagrophytes* (*T. mentagrophytes*) [15].

On the other hand, coevolution between the host and fungus is of the upmost importance as, depending on its adaptation, the dermatophytes can efficiently evade the host’s immune response. It is worth mentioning that dermatophytes as a group share physiologic and morphologic features among them, although they present nutritional and enzymatic differences [1,2,3,4]. For example, anthropophilic dermatophytes are able to inhibit the skin’s inflammatory response, and thus, they can establish chronic infections in a very efficient manner, in contrast to geophilic or zoophilic dermatophytes that produce an exaggerated inflammatory response [3,4,16].

In order to establish infection, the fungi must overcome a series of obstacles to be able to adhere, grow, and invade the host, for which their virulence factors are important [2,3,17]. Phenotypic virulence factors are genetically defined, being fundamentally structural and enzymatic. It should be noted that enzymes can be generated from an inflammatory response to regulate the innate immunity of the host [12,13].

Among the main enzymes recognized as virulence factors, hydrolases such as lipase and phospholipase have been reported to be produced by *T. rubrum* strains [18]. As for lipases, these enzymes act in the transesterification or hydrolysis of long-chain triglycerides, just as phospholipases hydrolyze the ester bonds of glycerophospholipids in the membranes of host cells [19].

On the other hand, proteases are the most studied virulence factors in mycoses because they are fundamental for acquiring and absorbing nutrients in the adhesion stage, as well as during germination and tissue colonization [18]. Among the main proteases are hemolysins, which are exotoxins capable of lysing red blood cells and nucleated cells [18,19,20], and elastases whose hydrolyzation generates a quick dissolution of elastic fibers [21]. Previous studies have reported the generation of proteases, phospholipases, hemolysins, and elastases by *T. rubrum* [22,23]. Therefore, the objective of this study was the genotypic identification of clinical isolates of *T. rubrum* in order to determine the production of the main phenotypic virulence factors associated with this pathogen responsible for different types of dermatophytosis in Mexican patients.

## 2. Materials and Methods

Twenty analyzed samples of *T. rubrum* were obtained from patients with clinical manifestations in the topographical regions of the soles, palms, head, nails, and interdigital regions of the feet, who were treated between June 2021 and June 2023 in the Mycology section of the General Hospital “Dr. Manuel Gea González” in Mexico City. Patients with *tinea capitis* had diffuse alopecia, scaling, and small pseudoalopecic plaques with blackheads; those with onychomycosis (*tinea unguium*, DSO, TDO, and LDSO) were generally observed with hyperkeratosis, thickening, and a yellowish, brown, or grayish color; patients with tinea pedis presented with scales, maceration, cracks, and fissures, and, in some cases, hyperkeratosis. The samples were taken by shaving using a No. 15 scalpel. This study was approved by the Research Ethics Committees of the Hospital Regional de Alta Especialidad de Ixtapaluca, HRAEI (NR- CEI-HRAEI-38-2021).

A clinical diagnosis was made by a dermatologist who also obtained the scales for the KOH mount and culture in Mycosel agar. Phenotypic identification was corroborated by partial sequencing of the β-tubulin gene using the primers Bt2a GGTAACCAAATCGGTGCTGCTTTC and Bt2b CCCTCAGTGTAGTGACCCTTGGC. The obtained sequences were deposited in GenBank (accession no. PV100845-PV100864).

After 4 weeks, the colonies that grew were transferred to specific agars to analyze the production of phenotypical virulence factors. These studies were carried out in triplicate for each strain on each of the following media (Table 1).

All preparations were sterilized in an autoclave at 121 °C and with 15 pressure pounds. They were evaluated twice a week for 5 weeks [24,25].

The database was recorded in Excel, registering culture, patients age, sex, place of birth, place of residence, occupation, comorbidities, localization of lesions, evolution, and clinical diagnosis. For phenotypical virulence factor evaluation, shift areas in the cultures as well as the growth halo were measured. After that, enzyme activity was calculated by the precipitation zone (Pz) with the following formula: Pz = (colony diameter)/(colony diameter with zone of precipitation). The isolates were, therefore, classified into ultra-producers (<0.35), high producers (Pz = 0.35–0.5), moderate producers (Pz = 0.51–0.74), low producers (Pz = 0.75–0.9), and non-producers (Pz = 1) [26,27].

### Statistical Analysis

For data analysis, descriptive and bivariate statistics were applied (Prism, GraphPad version 9, BOSTON, MA 02110, USA). For quantitative variables, the Kolmogorov–Smirnov normality test was performed (*n* > 30), and the median and interquartile ranges (RIQ) 25, 75 were used as the data had a free distribution. To determine whether there were differences between the activity of each of the enzymes (hemolysin, esterase, phospholipase, and proteinase) in the isolates, the Kruskal–Wallis test was performed. A *p* > 0.05 was taken into account to determine statistically significant differences.

## 3. Results

**Figure 1:**
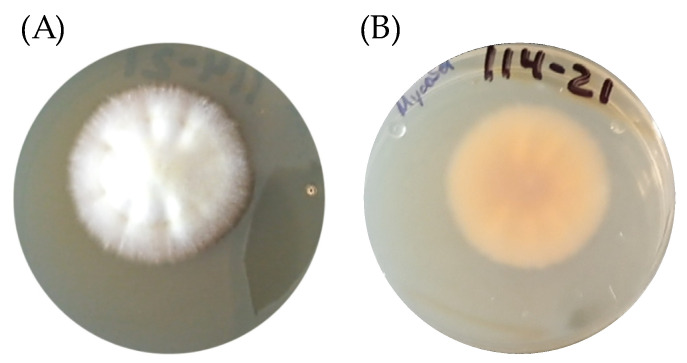
Macroscopic characteristic of *T. rubrum* in Mycosel^®^ agar. (**A**) Obverse and (**B**) Reverse of the Petri dish.

In Figure 1, we observe the macroscopic characteristics of *T. rubrum*: white mycelia, powdery, and surrounded by a reddish halo in Mycosel^®^ agar (Figure 1).

In Table 2, the sociodemographic and clinical characteristics of the studied population are described. The most prevalent form presented was *tinea unguium* (*n* = 6), distal subungual onychomycosis (DSO) and total dystrophic onychomycosis (TDO) (*n* = 4), *tinea pedis* (*n* = 3), *tinea capitis* (*n* = 2), and lateral distal subungual onychomycosis (LDSO) and TDO/tinea pedis (*n* = 1).

**Figure 2:**
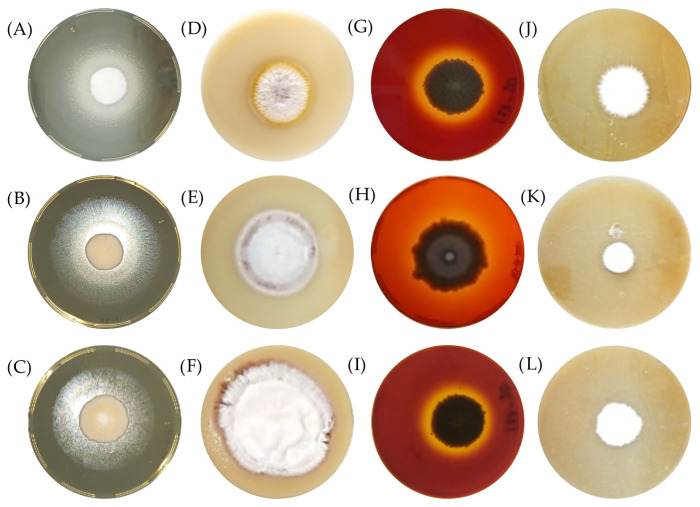
Macroscopic characteristics of *T. rubrum* cultures in different media for the production test of lipase (**A**–**C**), phospholipase (**D**–**F**), hemolysins (**G**–**I**), and elastase (**J**–**L**).

In relation to fungal growth, all strains of *T. rubrum* studied showed growth in the test culture medium except strain 114-21 in the lipase test medium (Table 3). Figure 2 shows the macroscopic characteristics and production of the different virulence factors studied of the *T. rubrum* strains. In all the culture test media, powdery and circular colonies were observed; white mycelia with a yellow or reddish coloration, elevated, with iridescent shifts of the medium (lipase: Figure 2A–C; phospholipase: Figure 2D–F; hemolysins: Figure 2G–I; elastase: Figure 2J–L). Notably, in phospholipase medium tests, in some cases, an invasive growth of the fungi was observed. As for enzyme production tests, in all test culture media (*n* = 20) for phospholipase and hemolysins, as well as in the remaining 19 strains for the lipase medium test, there was a shift in color in the test culture medium, which indicates the production of enzymes by the strains (Figure 2 and Table 3). It is noteworthy that elastase production was observed in only seven strains (79-20, 80-21, 88-21, 113-A-21, 127-20, 31593-D, and 30354-D) (Table 3).

**Figure 3:**
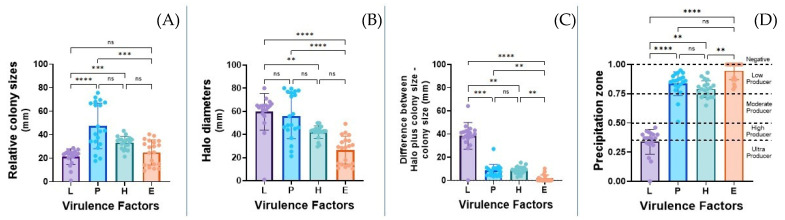
Determination of the different phenotypic virulence factors (L, P, H, and E) of *T. rubrum*, taking into account the (**A**) growth size of the colony in mm, (**B**) size of the colony plus degradation halo in mm, (**C**) width of the degradation halo in mm, and (**D**) the precipitation zone observed as an ultra-producer of lipase, low producer of phospholipases and hemolysins, and between low and no producer of elastase. Ultra-producers (<0.35), high producers (Pz = 0.35–0.5), moderate producers (Pz = 0.51–0.74), low producers (Pz = 0.75–0.9), and non-producers (Pz = 1). ** *p* < 0.01, *** *p* < 0.001, **** *p* < 0.0001.

Figure 3 presents the (A) colony size, (B) degradation halos’ diameters in all the agars employed, (C) difference observed between the colony size plus the halos’ diameters and colony size, and (D) the precipitation zone analyzed in the study. It was observed that the strains in phospholipase agar had a statistically greater growth (47.43 ± 19.37 mm) than those in hemolysin (33.21 mm ± 5.33), lipase (22.22 ± 6.77 mm), and elastase (24.80 ± 10.58 mm) agars (Figure 3A). Likewise, there was a significantly smaller size of the halo diameter of elastase (26.51 ± 11.95 mm) in comparison to lipase (59.51 ± 16.00 mm) and phospholipase (55.97 ± 19.60 mm). Additionally, the halo diameter of hemolysin was of a significantly reduced size (42.01 ± 5.49 mm) compared to lipase. No other statistically significant difference was observed (Figure 3B). Regarding the difference between the halo plus colony size and the colony size alone, a greater significant difference was observed in the lipase test medium (38.35 ± 11.57 mm) compared to the other media tested, and it was statistically lower in elastase (1.78 ± 2.92 mm) compared with phospholipase and hemolysin (phospholipase = 8.54 ± 5.27 mm and hemolysin= 8.81 ± 3.18 mm) (Figure 3C).

When comparing the four virulence factors, greater expression of lipase was observed, followed by phospholipase, hemolysins, and elastase. Regarding the precipitation zone (Figure 3D), it was observed that most of the *T. rubrum* strains were between high and ultra-lipase producers (0.3390 ± 0.1063); most of the strains were also considered low producers of phospholipase and hemolysins. It is important to note that in the case of elastase, most of the strains (*n* = 13) were non-producers (Figure 3D).

**Figure 4:**
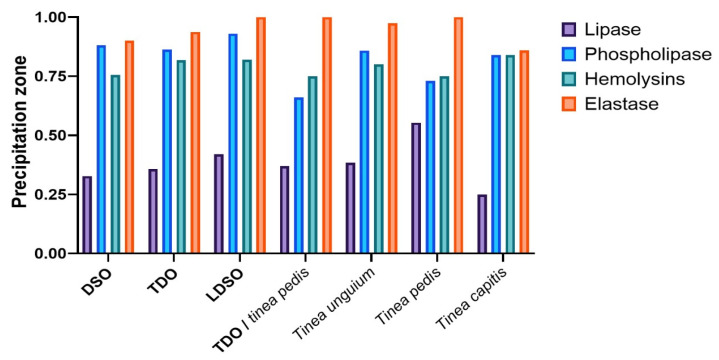
Graphical representation of the precipitation zones of the phenotypic virulence factors analyzed (purple lipase, sky blue phospholipase, green hemolysins, and orange elastase), observed according to the dermatophytosis presented in each patient. A smaller area of precipitation produced by lipase was observed in all clinical presentations, indicating ultra-production of this enzyme, except in *tinea pedis* where it was classified as a moderate producer of the enzyme. On the other hand, it was observed that elastase in practically all clinical presentations was greater than 0.75, indicating low production of it. Ultra-producers (<0.35), high producers (Pz = 0.35–0.5), moderate producers (Pz = 0.51–0.74), low producers (Pz = 0.75–0.9), and non-producers (Pz = 1).

Figure 4 shows changes in the production of the different phenotypic factors studied (lipase, phospholipase, hemolysins, and elastase) in the clinical strains analyzed. It was observed that in all cases of dermatophytosis, there was ultra-production of lipase, low production of phospholipase and hemolysins, and low or no production of elastase. It is important to mention that regarding the TDO/tinea pedis sample, phospholipase production was considered moderate.

## 4. Discussion

Currently, the importance of dermatophyte virulence factors for invasion, colonization, growth, and dissemination to host tissues, as well as drug resistance in some cases, has been evidenced [18,28]. The main etiological agent of dermatophytosis in humans worldwide to date is *T. rubrum* [18]. There is little evidence in Mexico of the virulence factors of *T. rubrum*. Due to the above, the study aims to verify if the different phenotypic virulence factors of *T. rubrum* correlate to clinical presentation. It is recognized that *T. rubrum* is an anthropophilic fungus well-adapted for keratinized tissues by synthesizing several enzymes [1,18,29]. Several authors have studied genes involved in cell wall biosynthesis that could encode virulence factors when exposing *T. rubrum* to different drugs (undecanoic acid, acriflavine, trans-calcona, etc.) and environmental stress factors [26]. Among the most studied virulence factors in *T. rubrum* are lipase, phospholipase, hemolysins, and elastase [1,18,19,20,29,30,31].

Fungi that have adapted to the surface of human skin are found in an environment devoid of carbohydrates, so they have developed the ability to metabolize fatty acids [32]. Some of these lipid components of the skin are squalene, wax monoesters, and triglycerides, with small amounts of cholesterol and cholesterol esters [33]. As mentioned above, lipases produce transesterification in the ester bonds of water-insoluble substrates, as well as in hydrolysis. They can produce diacylglycerol, monoacylglycerol, glycerol, and free fatty acids [34,35]. The lipase produced by dermatophytes is of importance as it allows it to parasitize the skin. This is achieved by using lipids as a primary source of carbon and nutrients in the germination stage and, subsequently, penetrating the lower layers of the epidermis that contain a greater number of proteins [36]. In the present study, although one strain of *T. rubrum* did not present a lipase degradation halo (*n* = 19, 95%), the rest of them considerably exhibited it. This was evident in all clinical forms when there was ultra-production of lipase, except in *tinea pedis* in which moderate production was observed. In agreement with the present study, López Martínez et al., when describing the relationship between the clinical forms of dermatosis and the presence of virulence factors, reported an important expression of lipase (*n* = 38, 80.8%) and DNase (*n* = 46, 97.8%) in 47 strains of *T. rubrum* [19].

As for phospholipases, these enzymes allow fungi and bacteria to have a high virulent potential [37]. Likewise, it is important to clarify that phospholipase activity does not have hemolytic activity by itself, but hemolysins are necessary to produce it [20]. It should be noted that in the case of hemolysins, as happens in several bacteria, these enzymes are one of the main virulence factors that mediate the severity of infections in vertebrates and the loss of such activity produces avirulence of these bacteria [38,39]. However, unlike what is observed in bacteria, fungi have a slow growth rate, which means that the study of virulence factors such as hemolysins must be carried out over a longer period [20]. In dermatophytes, hemolysins have been directly related to the severity and chronicity of the pathology [19,20].

In the present study, phospholipase and hemolysin production was observed in all the samples (*n* = 20, 100%). Nevertheless, the expression of both enzymes was low in concentration and low production. It is important to mention that, although in all clinical presentations both phospholipases and hemolysins were classified as low producers, moderate production of phospholipases was observed in the TDO/tinea pedis sample. Differential enzyme production has also been observed with subtilisins and metalloproteases, where SUB3, SUB4, and MEP4 genes show higher expression when *T. rubrum* is grown on media containing keratin, elastin, or collagen compared to those containing only glucose [40]. It is likely that in patients with TDO, there are factors that positively regulate genes encoding for phospholipases and hemolysins, as well as other endo- and exoproteases. These have been reported in the regulation of *T. rubrum* proteases in keratin-added cultures, although not all keratin-induced proteases play a role during infection [41]. In addition, it is important to note that environmental factors, such as pH, also play a crucial role in the regulation of gene expression [42,43]. Schaufuss and Steller studied the presence of hemolysins in different *Trichophyton* species. In the case of *T. rubrum*, they observed cottony colonial morphology growth on CBAP agar after 7 days. Interestingly, the authors concluded that there was production of two different cytolytic factors since *T. rubrum* strains were surrounded by a bizonal lytic effect (zone of complete lysis and zone of incomplete lysis) after cultivation in CBAP with sheep, bovine, and horse blood [20]. In disagreement with our results, López Martínez et al., in their study with 47 strains of *T. rubrum*, reported low production of hemolysins, only existing in 6 of them (12.8%) [19]. It is important to mention that, in the present study, even though all strains showed production of phospholipase and hemolysins at low concentrations, this could be because no further production was necessary for its establishment as a pathogen. It is known that dermatophytes generally affect the stratum corneum and very rarely cause complex, severe, and invasive infection [42]. As mentioned above, phospholipases allow establishment of the fungus through the degradation of glycerophospholipids, so this concentration was sufficient for its establishment. On the other hand, since an invasive process is not necessary, it is not important to present high concentrations of hemolysins because there is no contact with blood.

Regarding elastase, of the total of 20 studied strains, 13 did not produce it (65%). As previously mentioned, elastase has proteolytic activity for the dissolution of elastin, which is shared by many dermatophytes and some fungi that are capable of subcutaneous infections [21]. As for this enzyme, in all the clinical forms studied, low production of this was observed. The present results are consistent with studies conducted by Hopsu-Havu et al. and Riporn and Varadi in which they reported zero activity of this enzyme after 7 and 14 days, respectively [21,44]. However, in disagreement with López Martínez et al., they reported a wide expression of this enzyme being observed in up to 78.7% (*n* = 37) of the samples studied (*n* = 47) [19].

### Limitations

The main limitations of this study on phenotypic virulence factors of *T. rubrum* were as follows: First, the use of only 20 strains of the fungus obtained from a single center, which creates bias in terms of their location and characteristics. Also, since there are a limited number of samples with heterogeneous comorbidities and, taking into account that they are pathologies with diverse characteristics (metabolic, immunocompromised, and neurological), it is impossible to subdivide between groups and to have a considerable number that allows us to make associations between pathologies and the different enzymes studied. On the other hand, even though the strains were identified genotypically, only phenotypic studies were performed in terms of the production of the enzymes analyzed. Another point that could be important to address in the following studies is the search for an association between the clinical characteristics of the severity of the mycosis, as well as the pharmacological resistance of these strains with the presence and concentration of these proteases.

## 5. Conclusions

*T. rubrum* is the most common etiological agent in the diagnosis of any of the different types of ringworm. For this, virulence factors are important for adhesion, growth, and invasion in infected tissues. Regarding lipase, although not present in all strains, in most of them, there was ultra-production of this enzyme. On the other hand, even though all the strains expressed hemolysins and phospholipases too, they were considered low producers. Elastase was the least expressed virulence factor in these strains.

## Figures and Tables

**Table 1:** Formulation of agars used for the analysis of enzyme production in *T. rubrum* strains.

Media Type	Composition	Amounts
Blood agar for hemolysines evaluation	Casein peptone	10 g
	Yeast extract	3 g
	Heart extract	3 g
	Sodium chloride	5 g
	Bacteriologic agar 15 gr, Defibrinated lambs blood	15 g
		50 mL
	Distilled water	1000 mL
Agar for elastase evaluation	Bacteriologic agar	20 g
	Casein peptone	17 g
	Soy peptone	3 g
	Dextrose	2.5 g
	Sodium chloride	5 g
	Dipotassic phosphate	2.5 g
	Distilled water	1000 mL
	Bovine elastine	3 g
Agar for phospholipase evaluation	Peptone	10 g
	Dextrose	20 g
	Sodium chloride	57.3 g
	Calcium chloride	0.5 g
	Agar	20 g
	Sterile egg yolk	50 g
	Distilled water	1000 mL
Agar for lipase evaluation	Peptone 10 gr,	10 g
	Sodium chloride 5 gr,	5 g
	Calcium chloride 0.1 gr,	g
	Agar 20 gr,	20 g
	Tween 20	10 mL
	Distilled water	1000 mL

**Table 2:** Description of the sociodemographic and clinical characteristics of the study population.

Strain Number	Age (Years)/Sex	Occupation	Origin/Residence	Comorbidities	Localization	Evolution Time	Diagnosis
**41-21**	28/F	Housewife	Mexico City/Mexico City	NA	Toenails	5 months	DSO
**43-21**	28/M	Assistant Manager	Mexico City/Mexico City	NA	Toenails	1 year	DSO
**48-21**	35/F	Housewife	Mexico City/Mexico City	NA	Toenails	1 year	*Tinea unguium*
**49-21**	19/F	Student	Mexico City/Mexico City	NA	Toenails	6 months	*Tinea unguium*
**79-20**	43/F	Housewife	Puebla/Puebla	Breast Cancer/Diabetes Mellitus	Toenails	1 year	DSO
**80-21**	41/M	Tailor	Mexico City/Mexico City	Lymphoma no Hodgkin	Toenails	5 years	DSO
**88-21**	50/M	Bus driver	Mexico City/Mexico City	HIV	Toenails	1 year	TDO
**90-B-21**	13/M	Student	Mexico City/Mexico City	NA	Toenails	4 years	LDSO
**102-B-21**	51/M	Taxi driver	Mexico City/Mexico City	HIV/Epilepsy	Toenails and interdigital injuries	4 months	TDO/*tinea pedis*
**103-21**	34/F	Housewife	Mexico City/Mexico City	Dermatomyositis	First right foot ortho	2 years	TDO
**113-A-21**	29/M	Student	Mexico City/Mexico City	HIV	Toenails	1½ years	TDO
**114-21**	34/M	Government employee	Mexico City/Mexico City	NA	Soles of the feet	6 months	*Tinea pedis*
**116-21**	34/M	Government employee	Mexico City/Mexico City	NA	Toenails	1 year	*Tinea unguium*
**119-21**	62/M	Unemployed	Mexico City/Mexico City	Diabetes mellitus	Left toenail	1 year	*Tinea unguium*
**122-20**	53/M	Government employee	Tamaulipas/Tamaulipas	NA	Soles of the feet	1 year	*Tinea pedis*
**124-20**	26/M	Security guard	State of Mexico/Mexico City	NA	Soles of the feet	1 year	*Tinea pedis*
**127-20**	22/F	Student	Mexico City/Mexico City	NA	Toenails	1 year	*Tinea unguium*
**139-A-20**	71/F	Real estate advisor	Veracruz/Mexico City	Hypothyroidism	Toenails	15 years	TDO
**31593-D**	7/M	Elementary student	Mexico City/Mexico City	NA	Scalp	3 weeks	*Tinea capitis*
**30354-D**	5/M	Elementary student	Mexico City/Mexico City	NA	Scalp	1 month	*Tinea capitis*

No patients received prior antifungal treatment. F = female; M = male; NA = not available; HIV = human immunodeficiency virus; DSO = distal subungual onychomycosis; TDO = total dystrophic onychomycosis; LDSO = lateral distal subungual onychomycosis. It is observed that most of the patients did not have previously reported comorbidities (60%). Most of the samples had a maximum of one year of evolution (65%).

**Table 3:** Measures of phenotypic virulence factors of *T. rubrum*.

Strain	Colony Size				Degradation Halo Size				Difference				Precipitation Zone			
	L	P	H	E	L	P	H	E	L	P	H	E	L	P	H	E
**114-21**	0	17.6	35	31	0	21.3	43	31	0	3.7	8	0	0.00	0.83	0.81	1.00
**30354-D**	8.3	47	27	9.3	50	57.8	30.7	10.5	41.7	10.8	3.7	1.2	0.17	0.81	0.88	0.89
**43-21**	15.8	65	26.4	30	80	74	37.3	30	64.2	9	10.9	0	0.20	0.88	0.71	1.00
**103-21**	20	31.9	21.3	14.1	64.7	37	29.6	14.1	44.7	5.1	8.3	0	0.31	0.86	0.72	1.00
**119-21**	21.6	68.1	35.8	12	68.3	72.9	43	12	46.7	4.8	7.2	0	0.32	0.93	0.83	1.00
**79-20**	21.4	43.5	31.1	39	64.6	48.2	42	49.2	43.2	4.7	10.9	10.2	0.33	0.90	0.74	0.79
**31593-D**	13.5	21.9	26.4	11	41.3	25.3	32.8	13.3	27.8	3.4	6.4	2.3	0.33	0.87	0.80	0.83
**113-A-21**	23.4	75.6	40.4	36.4	66.5	80	45	41.6	43.1	4.4	4.6	5.2	0.35	0.95	0.90	0.88
**41-21**	23.8	67.4	35.7	33.3	65	79.6	45	33.3	41.2	12.2	9.3	0	0.37	0.85	0.79	1.00
**88-21**	22.7	65	38.7	24.6	61	76	41.6	28.4	38.3	11	2.9	3.8	0.37	0.86	0.93	0.87
**102-B-21**	23.3	19.6	33.9	11	63.4	29.9	45	11	40.1	10.3	11.1	0	0.37	0.66	0.75	1.00
**116-21**	24	37.5	43.3	18	64.9	46	49.9	18	40.9	8.5	6.6	0	0.37	0.82	0.87	1.00
**127-20**	23.5	66.7	31	25	63.4	77.3	43	28.9	39.9	10.6	12	3.9	0.37	0.86	0.72	0.87
**48-21**	28.6	42.8	35	40.3	71	46.4	42.9	40.3	42.4	3.6	7.9	0	0.40	0.92	0.82	1.00
**122-20**	24.7	28.7	28.3	29.3	61	55.8	43.7	29.3	36.3	27.1	15.4	0	0.40	0.51	0.65	1.00
**139-A-20**	24.4	34.3	35.8	34.7	61.4	44	49.8	34.7	37	9.7	14	0	0.40	0.78	0.72	1.00
**80-21**	26.5	69.6	33.8	32	64.7	78	43.2	39.6	38.2	8.4	9.4	7.6	0.41	0.89	0.78	0.81
**90-B-21**	27.3	71.7	36	35	65	77	43.7	35	37.7	5.3	7.7	0	0.42	0.93	0.82	1.00
**124-20**	24.9	40.5	37	14.9	58	47.9	46.8	14.9	33.1	7.4	9.8	0	0.43	0.85	0.79	1.00
**49-21**	25.6	34.2	32.2	15	56	45	42.2	15	30.4	10.8	10	0	0.46	0.76	0.76	1.00

L = lipase; P = phospholipase; H = hemolysins; E = elastase.

## Data Availability

The original contributions presented in this study are included in the article. Further inquiries can be directed to the corresponding author.
